# Analysis of the diagnostic and prognostic value of miR-9-5p in carotid artery stenosis

**DOI:** 10.17305/bjbms.2021.5545

**Published:** 2021-12

**Authors:** Hongxin Liu, Juan Zhou, Wei Jiang, Feng Wang

**Affiliations:** 1 Department of Neurology, Yidu Central Hospital of Weifang, Weifang, China; 2 Department of Pediatrics, Yidu Central Hospital of Weifang, Weifang, China; 3 Department of Neurosurgery, Zhucheng People’s Hospital, Weifang, China

**Keywords:** MiR-9-5p, asymptomatic CAS, diagnosis, prognosis, cerebrovascular events

## Abstract

More and more evidence shows that microRNAs (miRNAs) play an important role in the diagnosis and prognosis of human diseases. In this study, we investigated the diagnostic value of miR-9-5p for asymptomatic carotid artery stenosis (CAS) and its predictive value for future cerebrovascular events within 5 years. A total of 88 asymptomatic CAS patients and 86 healthy individuals were recruited. The expression level of serum miR-9-5p was determined by quantitative real-time polymerase chain reaction (qRT-PCR). The diagnostic value of miR-9-5p in CAS was assessed by a receiving operator characteristic (ROC) curve. The predictive value of miR-9-5p for the occurrence of cerebrovascular events was evaluated by the Kaplan-Meier method. The serum level of miR-9-5p was significantly decreased in asymptomatic CAS patients. ROC curve had an AUC value of 0.910, with the sensitivity and specificity of 80.7% and 87.2% at the cut-off value of 0.72, respectively. A total of 25 patients had cerebrovascular events during the 5-year follow-up, including 3 strokes and 22 transient ischemic attacks (TIAs). Kaplan-Meier survival analysis revealed that the low expression level of miR-9-5p was an independent factor closely related to the occurrence of cerebrovascular events. Serum miR-9-5p could be used as a new biomarker for the diagnosis of CAS, and the low expression of miR-9-5p is associated with poor prognosis.

## INTRODUCTION

The carotid artery is a large vessel that carries blood from the heart to the head, neck, and face, and is one of the main blood vessels for the brain [1]. Carotid artery stenosis (CAS) is a common form of atherosclerotic vascular disease and is a major risk factor for cerebrovascular events [2]. CAS is mainly divided into symptomatic CAS and asymptomatic CAS [3]. In the early stages, most patients with CAS are asymptomatic and undetectable. Asymptomatic CAS also increased the risk of stroke by more than 3% in the 2^nd^ year (relative risk increased by more than 50%) [4]. Early diagnosis and timely intervention are extremely effective for the prevention and treatment of CAS. Therefore, it is necessary to search for a new biomarker to predict and diagnose the occurrence of asymptomatic CAS.

MicroRNAs (miRNAs) are a class of small, highly conserved non-coding RNA molecules, with approximately 22 nucleotides in length [5]. miRNAs are involved in the regulation of gene expression at the post-transcriptional level by degrading their target mRNA and/or inhibiting its translation [6-8]. In recent decades, the role of miRNA in the growth and development of tissues and the occurrence and development of diseases has been gradually recognized. For example, Chen et al. reported that miR-424-5p was involved in regulating the occurrence of hepatitis B virus-associated hepatocellular carcinoma [9]. Guo et al. found that overexpression of miR-129-5p in glioblastoma revealed an inhibitory effect on tumor cell proliferation and migration by targeting ZFP36L1 [10]. In the cardio-cerebrovascular system, miRNA maintained the growth of cardiomyocytes, angiogenesis, plaque formation, and lipid metabolism [11,12]. Abnormal expression of related miRNAs can be detected in the blood of patients with various cardio-cerebrovascular diseases, which makes them excellent candidates for non-invasive diagnostic biomarkers [11]. Yan et al. found that the expression of miR-503-5p was decreased in the serum of asymptomatic CAS patients, and the low expression of miR-503-5p significantly promoted the proliferation of vascular smooth muscle cells (VSMCs) [12]. A previous study showed that compared with normal carotid arteries, miRNA expression was significantly upregulated in carotid arteries with restenosis after stent implantation, including miR-17, miR-18a, miR-19a, miR-20a, and miR-92a [13]. MiR-9-5p, also commonly expressed as miR-9, acted as an oncogene in tumors [14]. Notably, downregulation of miR-9 has been shown to be functionally involved in the development of cardiovascular diseases, such as cardiac hypertrophy and heart failure [15]. However, the role of miR-9-5p in CAS is still unclear. Moreover, exploring the clinical significance of miR-9-5p in CAS will help us better understand its value in diagnosis and prognosis of CAS.

In the present study, we measured the expression level of miR-9-5p in CAS patients and healthy controls. Considering the difference in the expression of miR-9-5p in the two groups, we further evaluated the value of miR-9-5p in the diagnosis and prognosis of CAS patients.

## MATERIALS AND METHODS

### Study population and sample collection

This protocol follows the ethical principles of human research in the Helsinki Declaration and has been approved by the Ethics Committee of Yidu Central Hospital of Weifang. All individuals recruited for this study have signed an informed consent.

From January 2014 to April 2015, a total of 88 asymptomatic CAS patients were included in Yidu Central Hospital of Weifang, and the exclusion criteria were as follows: (1) Patients who suffer from atrial fibrillation, myocardial infarction, cardiomyopathy, and serious lung disease that may lead to stroke or arrhythmia and (2) patients with malignant tumors. CAS is defined as the stenosis of the extracranial internal carotid artery greater than 50%, and the degree of stenosis was evaluated by the North American symptomatic carotid endarterectomy trial method [16]. Asymptomatic status was assessed by reviewing patient history, performing a physical examination, and investigating the National Institutes of Health Stroke Scale [17]. In addition, 86 healthy volunteers were selected as the healthy control group, without the history of inflammation of metabolic diseases, malignant tumors, and cardiovascular and cerebrovascular diseases. All subjects underwent color Doppler ultrasound, and the degree of CAS was determined based on computed tomography angiography (CTA) measurements. Only those with normal Doppler ultrasonography or with internal CAS of less than 20% were included in the healthy control group. The sample size was estimated according to the data of our earlier pre-trial. a = 0.05, b = 0.1, the power = 0.9, and a dropout rate of 10%, at least 85 cases in each group were tested according to the two-tailed test. Therefore, the sample size of this study met the requirements. Fasting venous blood samples were collected from all subjects. After rapid centrifugation, serum samples were collected and stored in –80^o^C for later use. Demographic and clinical characteristics of patients were collected and recorded for further analysis.

### RNA extraction and quantitative real-time polymerase chain reaction (qRT-PCR) analysis

Total RNAs in serum were extracted using Trizol reagent (Invitrogen, Carlsbad, CA, USA) according to the product’s instruction. Briefly, after the cells were fully lysed, 200 mL of chloroform was added into the centrifuge tube, and the supernatant was obtained by centrifugation under the condition of 12,000 r/minutes at 4^o^C. The supernatant was mixed with cold isopropyl alcohol, and the precipitation was obtained after centrifugation. About 75% ethanol was added to the precipitate, mixed, and centrifuged. Finally, the precipitate was naturally dried to produce total RNA. The concentration and purity of RNA were detected by NanoDrop 2000c. If the ratio of OD260/OD280 was between 1.7 and 2.2, RNA purity is qualified. RNA integrity was detected by RNA 6000 Nano Kit, RIN <7 indicated that the integrity of the sample is available for experimental study. And then, total RNAs were reverse transcribed into cDNA using PrimeScript RT reagent kit (Takara, Tokyo, Japan). The expression level of miR-9-5p was detected using a miScript SYBR^®^ Green PCR kit (Qiagen GmbH, Germany) in Applied Biosystems 7300 Real-Time PCR System (Applied Biosystems, Foster City, CA) and was normalized by U6 using 2^−DDCt^ method. The forward primers of miR-9-5p and U6 were 5’-TCTTTGGTTATCTAGCTGTATGA-3’ and 5’-CTCGCTTCGGCAGCACA-3’. The reverse primers of miR-9-5p and U6 were the universal primers of the kit. qRT-PCR conditions were as follows: 1 cycle of 95^o^C for 5 minutes and 40 cycles of 95^o^C for 10 seconds, 60^o^C for 25 seconds, and 72^o^C for 35 seconds.

### Follow-up analysis

All the patients participated in a 5-year follow-up study. The occurrence of strokes, transient ischemic attack (TIA), or cardiovascular-related death were defined as primary cerebrovascular endpoint events, and the patients who died from other unrelated causes were excluded from the follow-up analysis.

### Statistical analysis

The data were analyzed using SPSS 21.0 software (SPSS Inc., Chicago, IL) and GraphPad Prism 7.0 software (GraphPad Software, Inc., USA). Chi-square test was used to compare categorical variables between groups, and continuous variables were compared between groups using the Student’s *t*-test. The value of miR-9-5p on predicting cerebrovascular endpoint events was analyzed by Kaplan–Meier method and Cox regression analysis. The value of *p* <0.05 was considered statistically significant.

## RESULTS

### Clinical data comparisons

The demographic characteristics and clinical data of healthy control group and asymptomatic CAS patient group in this study are shown in Table 1. It was found that there was no significant difference in age, gender, body mass index (BMI), total cholesterol, high-density lipoprotein, low-density lipoprotein, triglycerides, and fasting blood glucose between the healthy control and asymptomatic CAS groups (*p* > 0.05). However, the level of diastolic blood pressure (DBP) and systolic blood pressure (SBP) in asymptomatic CAS group was significantly higher than in the healthy control group (*p* < 0.05).

**TABLE 1:**
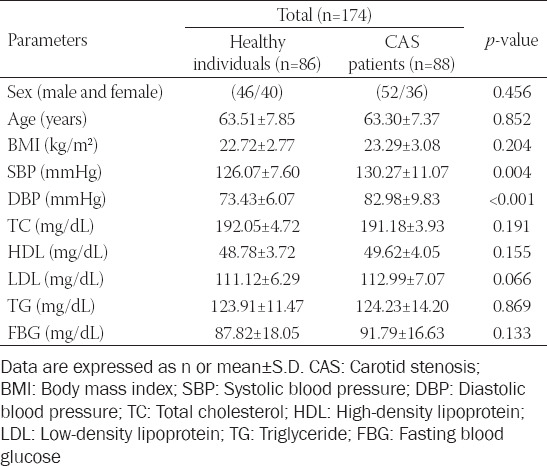
Clinical data of the study population

### Expression level of serum miR-9-5p in CAS patients

**FIGURE 1:**
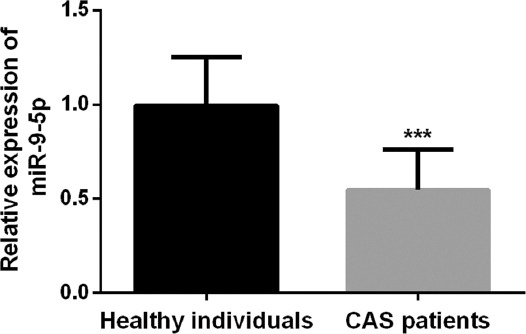
The expression level of serum miR-9-5p was significantly decreased in asymptomatic carotid artery stenosis patients compared with healthy controls (***p < 0.001).

The qRT-PCR results showed that the expression level of serum miR-9-5p in the asymptomatic CAS group was significantly lower than that in the healthy control group (Figure 1, *p* < 0.001), which indicated that serum miR-9-5p was associated with the occurrence of asymptomatic CAS.

### Receiving operator characteristic (ROC) curve analysis

**FIGURE 2:**
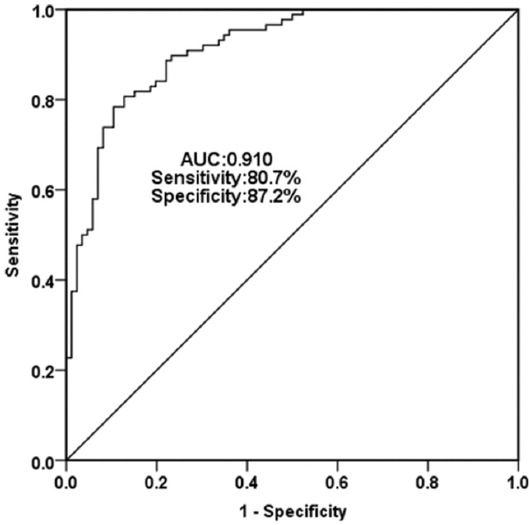
A receiving operator characteristic curve was established to evaluate the diagnostic value of miR-9-5p for asymptomatic carotid artery stenosis. The curve had an AUC value of 0.910 with the sensitivity of 80.7% and specificity of 87.2%.

The ROC curve was established to assess the diagnostic value of miR-9-5p for asymptomatic CAS according to the expression levels of serum miR-9-5p in all subjects. As shown in Figure 2, the curve had an AUC value of 0.910, the 95% CI was 0.869 to 0.952, with the sensitivity and specificity of 80.7% and 87.2%, respectively. The above results suggested that miR-9-5p has a high diagnostic accuracy in distinguishing asymptomatic CAS patients from healthy individuals.

### Predictive value of miR-9-5p in the occurrence of cerebrovascular events in asymptomatic CAS patients

According to the results of the 5-year follow-up analysis, Kaplan–Meier method was used to evaluate the predictive value of miR-9-5p level on cerebrovascular endpoint events in asymptomatic CAS patients. Based on the average expression level of miR-9-5p, the patients were divided into two groups: The high miR-9-5p expression group and the low miR-9-5p expression group. A total of 25 patients developed cerebrovascular endpoint events, including three strokes and 22 TIAs. Among the 25 patients, 18 patients were observed in low expression of miR-9-5p group and seven patients in high expression of miR-9-5p group.

**FIGURE 3:**
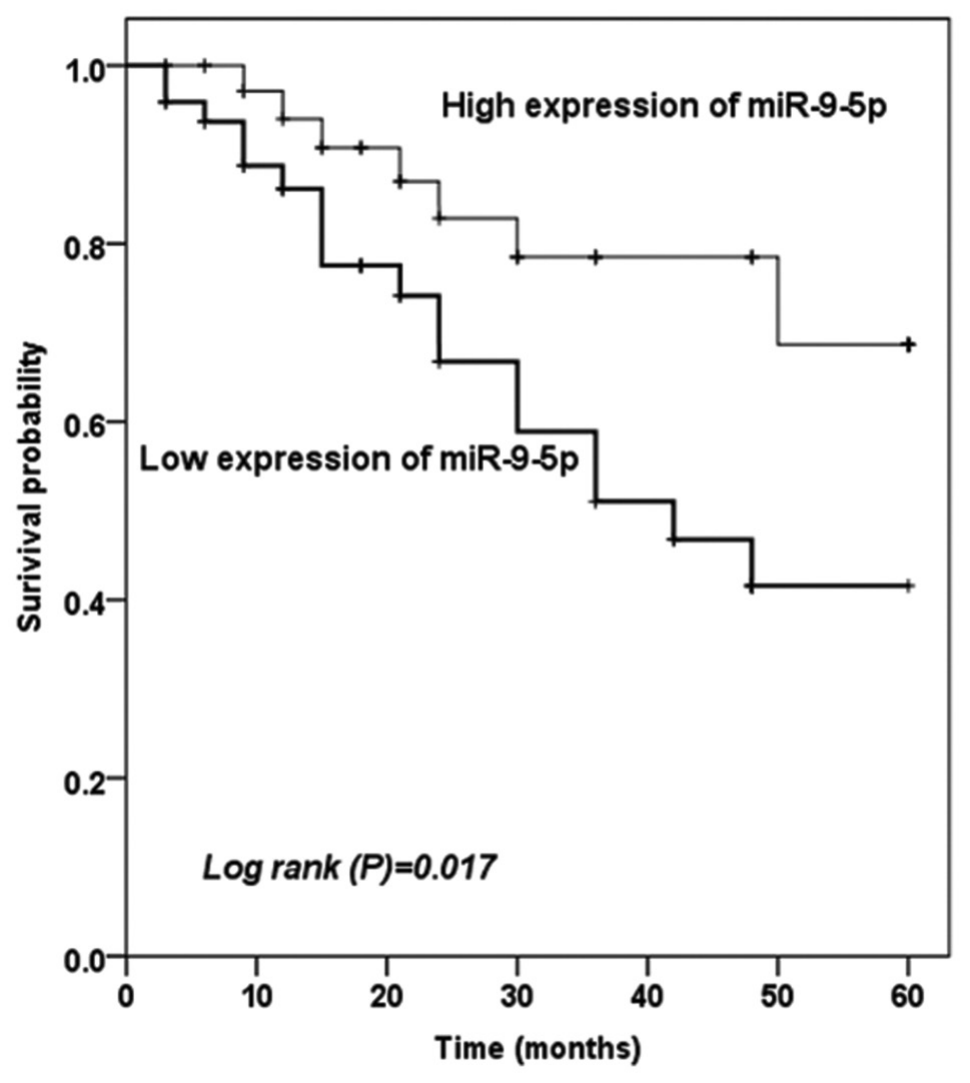
Kaplan–Meier survival curves for asymptomatic carotid artery stenosis patients with different expression levels of miR-9-5p, which could be observed that low miR-9-5p level was associated with the occurrence of cerebrovascular events.

Kaplan–Meier analysis revealed that patients with low miR-9-5p expression underwent more cerebrovascular events than those with high miR-9-5p expression (Figure 3, log rank *p* = 0.017). Multivariate Cox regression analysis was used to further determine the predictive variables for the occurrence of cerebrovascular events. As shown in Table 2, the risk of cerebrovascular endpoint events in the group with high miR-9-5p expression was 0.239 times than in the group with low miR-9-5p expression (95% CI: 0.087-0.652, *p* = 0.005), and the data suggested that low miR-9-5p level was independent factors for the occurrence of cerebrovascular events in asymptomatic CAS patients.

**TABLE 2:**
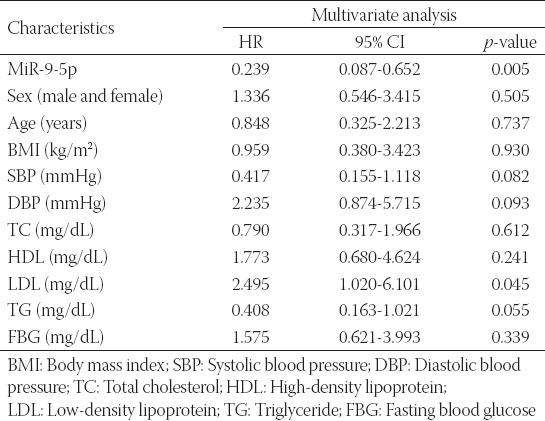
Multivariate Cox analysis of clinical characteristics in relation to overall survival

## DISCUSSION

This study aimed to analyze the diagnostic value of miR-9-5p for asymptomatic CAS and the predictive value of miR-9-5p for future cerebrovascular events in CAS patients by detecting the expression level of miR-9-5p in the serum of asymptomatic CAS. The results showed that serum miR-9-5p expression was significantly decreased in asymptomatic CAS patients compared with healthy controls. ROC results revealed that serum miR-9-5p had a high diagnostic accuracy in asymptomatic CAS patients. In addition, we investigated and recorded the predictive value of miR-9-5p in the occurrence of cerebrovascular events in asymptomatic CAS patients through a 5-year follow-up survey. We found that asymptomatic CAS patients with low expression of serum miR-9-5p had more cerebrovascular events, and multivariate COX regression analysis suggested that miR-9-5p might be an independent factor in predicting the occurrence of cerebrovascular events.

In recent years, due to the high incidence of vascular risk factors such as aging population, hypertension, diabetes, hypercholesterolemia, and smoking, the incidence of asymptomatic CAS has been increasing year by year [18]. Patients with asymptomatic CAS can develop from simple carotid media thickening to symptomatic CAS, which is a known risk factor for ischemic cerebrovascular disease [19]. Based on the noninvasive nature of circulating miRNAs in serum, the diagnostic value of miRNAs in cardio-cerebrovascular diseases has attracted extensive attention. A prospective study by Rafal et al. showed significant differences in miRNA expression profiles between symptomatic and asymptomatic CAS patients [20]. Many miRNAs have been shown to be abnormally expressed in human carotid plaques, such as miR-127 and miR-125a. These miRNAs may be involved in the proliferation and rupture of carotid plaques, and thus have a certain influence on CAS [21]. Studies have shown that miRNA was involved in the regulation of physiological functions of VSMCs. A study reported that miR-145, which is closely related to atherosclerosis, is significantly downregulated in the serum of CAS patients, and it inhibited the development of CAS by suppressing the proliferation and migration of VSMC [22]. Another study by Chen et al. reported that the expression of miR-214 was increased in plasma, and the overexpression of miR-214 played a role in the regulation of VSMC angiogenesis, proliferation, and senescence in CAS patients [6]. The above evidence illustrated the important role of miRNAs in the occurrence and development of CAS from the perspective of the pathogenesis of CAS.

MiR-9-5p is an ancient and highly conserved gene. In mammals, miR-9-5p is encoded by three genes: miR-9-1, miR-9-2, and miR-9-3, while in humans, these genes are located on chromosome 1q22, 5q14.3, and 15q26.1, respectively [23,24]. The previous studies have shown that miR-9-5p is involved in the regulation of proliferation, migration, and invasion of tumor cells, such as acute myeloid leukemia, non-small cell lung cancer [25], and prostate cancer [26]. In some recent studies, miR-9-5p has been found to be involved in the regulation of the development of cardio-cerebrovascular diseases. Wei et al. found that the expression level of miR-9-5p was significantly downregulated in the mouse model of ischemic brain injury. At the same time, the infusion of miR-9-5p mimics in mice not only reduced the cerebral infarct size but also reduced the apoptosis of brain neurons [27]. Zhang et al. reported that miR-9 expression was reduced in a mouse model of AS, and miR-9 delayed the development of AS by inhibiting SDC2 and the FAK/ERK signaling pathway [28]. In this study, after comparing the clinical data between CAS patients and healthy controls, it was found that the values of DBP and SBP in CAS patients were significantly higher than those in the healthy controls, suggesting that CAS patients had a higher risk of hypertension. CAS is closely related to the occurrence of hypertension. A study by Kontaraki et al. confirmed that the expression levels of miR-9 and miR-126 in serum of patients with hypertension were significantly lower than those of healthy people and were associated with hypertension prognosis and organ damage [29]. Taken together, in this study, we first confirmed the downregulation of miR-9-5p in the serum of asymptomatic CAS patients by qRT-PCR, which was consistent with the reduction of miR-9 in AS reported by Zhang et al. [28]. Then, the ROC curve revealed that miR-9-5p has the ability to distinguish CAS patients from healthy people. In the follow-up analysis, patients with low miR-9-5p expression were more likely to undergo cerebrovascular events. The Kaplan–Meier analysis was used to confirm that miR-9-5p could be used as an independent factor to predict cerebrovascular events. In a cross-sectional study of Przemysław et al., it was found that the independent risk factors for symptomatic carotid stenosis including diabetes, BMI, and chronic kidney disease. This study provides new insights into assessing whether patients with asymptomatic CAS develop plaque instability or convert to symptomatic CAS [30]. After analyzing the results of our study combined with the conclusions of the previous studies, we believe that miR-9-5p plays a significant role in regulating the occurrence and progression of CAS. Like most studies, the design of the current study is subject to limitations. During the follow-up period, lifestyle and other factors that may lead to poor prognosis were not included in the study, which may lead to bias of the results. Therefore, more studies are needed to confirm the current results.

## CONCLUSION

In conclusion, through experiments and data, we confirmed that the expression of serum miR-9-5p was decreased in asymptomatic CAS patients, suggesting that low level of miR-9-5p was related to the occurrence of CAS and miR-9-5p had the ability to be a candidate biomarker for the diagnosis of CAS. Furthermore, miR-9-5p may be an independent predictor of future cerebrovascular events in asymptomatic CAS patients. This study provides new ideas and insights for the role of miR-9-5p in CAS, but further in-depth exploration is needed to study the role and significance of miR-9-5p in the cardio-cerebrovascular diseases.
